# Psychometric evaluation of the modified 19-item Bengali version of WHOQOL scale using Rasch analysis: a cross-sectional study of a rural district in Bangladesh

**DOI:** 10.1186/s40359-020-00411-7

**Published:** 2020-05-01

**Authors:** Mohammed Nazim Uddin, Fakir M. Amirul Islam

**Affiliations:** 1 Department of Statistics, Data Science and Epidemiology; Faculty of Health, Arts and Design, Swinburne University of Technology, Hawthorn, VIC 3122 Australia; 2 Organisation for Rural Community Development (ORCD), Dariapur, Narail Bangladesh

**Keywords:** WHOQOL-BREF, Quality of life, Rasch analysis, Validation, Rural Bangladesh, Health related quality of life

## Abstract

**Background:**

This investigation aims to validate the psychometric properties of the modified 19-item Bengali version World Health Organization Quality of Life (WHOQOL) instrument in a typical healthy rural population in Bangladesh.

**Method:**

The cross-sectional investigation collected 300 adults aged 18–85 years from Narail, a rural district of Bangladesh using a multi-stage cluster random sampling technique. Face-to-face interviews were conducted between July and August 2018 using an Android phone installed with a mobile data collection application CommCare. SPSS version 25; IBM. and a Rasch analysis software RUMM2030 were used for analyses.

**Results:**

Results showed good overall fit, as indicated by a significant item-trait interaction with Bonferroni corrected *p* values, for physical (${\upchi}_{(20)}^2$ =32.13, *p* = 0.041), psychological (${\upchi}_{(16)}^2$ =14.93, *p* = 0.529), social (${\upchi}_{(16)}^2$ =12.62, *p* = 0.397), and environmental (${\upchi}_{(20)}^2$ =22.01, *p* = 0.339) domains. Item fit residual (IFR) values for all domains were within the desired limits, indicating no deviation from the expected relationship between the individual items and the rest of the items of the scale. Person fit residual (PFR) values also showed no person misfit among the samples, indicating item threshold are suitable for Rasch analysis. Reliability of the three domains of the 19-item WHOQOL scale was very good as indicated by a person separation index (PSI) = 0.873 and Cronbach’s Alpha (CA) = 0.881 for physical domain, PSI = 0.739 and CA = 0.746 for psychological domain, and PSI = 0.753 and CA = 0.781 for environmental domain. The social domain (PSI = 0.650 and CA = 0.669) had below acceptable reliability. All items in each domain had ordered thresholds except one item of the environmental domain. All four domains of the 19-item WHOQOL scale showed unidimensionality and was free from local dependency. Each domain also showed similar functioning for adults and older adults, males and females, no education and at least primary level of education, low and high socio-economic conditions.

**Conclusion:**

The 19-item modified WHOQOL scale is confirmed as an efficient screening tool for measuring QoL among healthy rural Bangladeshi people. The scale could be implemented more widely. In particular, validations are required for diseases-specific population in Bangladesh to measure the Health Related Quality of life (HR-QoL) assessments for people suffering from chronic or other diseases.

## Background

In recent times, there has been an increasing focus on estimations related to the quality of life (QoL) with significant outcomes in clinical settings made alongside assessments of the impact of the different interventions on QoL [1, 2]. QoL is affected by many components incorporating physical prosperity, psychological and emotional states, social associations, individual’s feelings and connection, and their relationship to the key features of their environment [3, 4]. In addition, social burden and distress can significantly affect wellbeing and influence overall QoL [4].

Regardless, most of the examinations of QoL have focused on the impact of chronic diseases which have been evaluated through the application of individual QoL estimation instruments [5–8]. Over the course of the last two decades, different tools have been developed to assess QoL [9]. However, these tools have been applied specifically to people with different diseases [10–12], with a few exceptions of their use in healthy people [13–15]. The WHOQOL-BREF has been one of the recognised instruments to measure QoL for general population [16–18]. The WHO Quality of life BREF (short form of the WHOQOL-100, which is 100 items) is a 26-item instrument that comprises items evaluated on a five-point Likert-type scale, is a culturally validated tool to measure QoL [19]. The WHOQOL-BREF is a multicultural QoL assessment scale available in 40 languages that covers four specific areas, physical, psychological, social relations and environmental health [20].

Bangladesh is a densely populated country with a population of 167 million people [21], with around 65% of living in rural areas [22]. The rural QoL index of Bangladesh is very low at 69.46 compared to the highest QoL index 198.57 which is in Denmark [23]. This statistic points to a major public health concern, particularly in rural areas. A Bengali version of the WHOQOL-BREF, translated from English to Bengali was used by Izutsu et al. [24] and Tsutsumi et al. [25], to measure QoL among young people and older adults living in the slum area of the capital city of Dhaka and some other rural areas of Bangladesh. Its validity was evaluated utilizing the Classical Test Theory (CTT) [24, 25]. The CTT has mainly three limiting properties, (i) the item and the person’s latent attributes cannot be estimated independently, and the item properties depend on a representative sample [26], (ii) each item is assumed to have equal contribution to the final score [27], and (iii) the response options are assumed to be equal for each item [28]. However, such constraints can usually be addressed using Rasch Measurement Theory [29].

Recently, Uddin et al. [30] validated the 26-item WHOQOL-BREF scale using a Rasch analysis technique in a rural district, Narail in Bangladesh and proposed a 19-item modified WHOQOL scale to measure QoL in general population. This consisted of; two overall items (overall QoL and general health), five items for the physical domain (instead of seven); four items for the psychological domain (instead of six); five items for the environmental domain (instead of eight). The social domain components remained the same. Four domains (physical, psychological, social and environmental) of the 19-item modified WHOQOL scale showed sufficient internal consistency, reliability, unidimensionality and similar features for different age-sex distributions. The 19-item modified WHOQOL scale was found to be more robust compared to the original 26-item, when evaluated under the rigorous assumptions of the Rasch measurement model [30].

The 19-item modified WHOQOL scale offers a more reliable way to measure QoL [30]. It is a useful screening tool for estimating QoL among the rural population of Bangladesh [30]. The 19-item scale also gives the additional advantage of reducing seven items from the original 26 items. The 19-item modified WHOQOL scale could be more useful in other rural settings to quantify the QoL with reasonably fewer items. Further checking of 19-item modified WHOQOL scale procedures utilizing Rasch examination requires extensive and thorough procedures that occupy a great deal of time, effort and continuous testing. However, there are potential advantages. This testing can broaden the individual project outcomes and support the research field. The culturally approved instrument of the 19-item modified WHOQOL scale could provide improvement to human service providers and could be applied in other developing nations with comparable socio-economic attributes.

Therefore, the present investigation plans to validate the four-domain of the 19-item modified WHOQOL scale in the rural healthy population in Bangladesh.

## Methods

### Study population

**Fig. 1:**
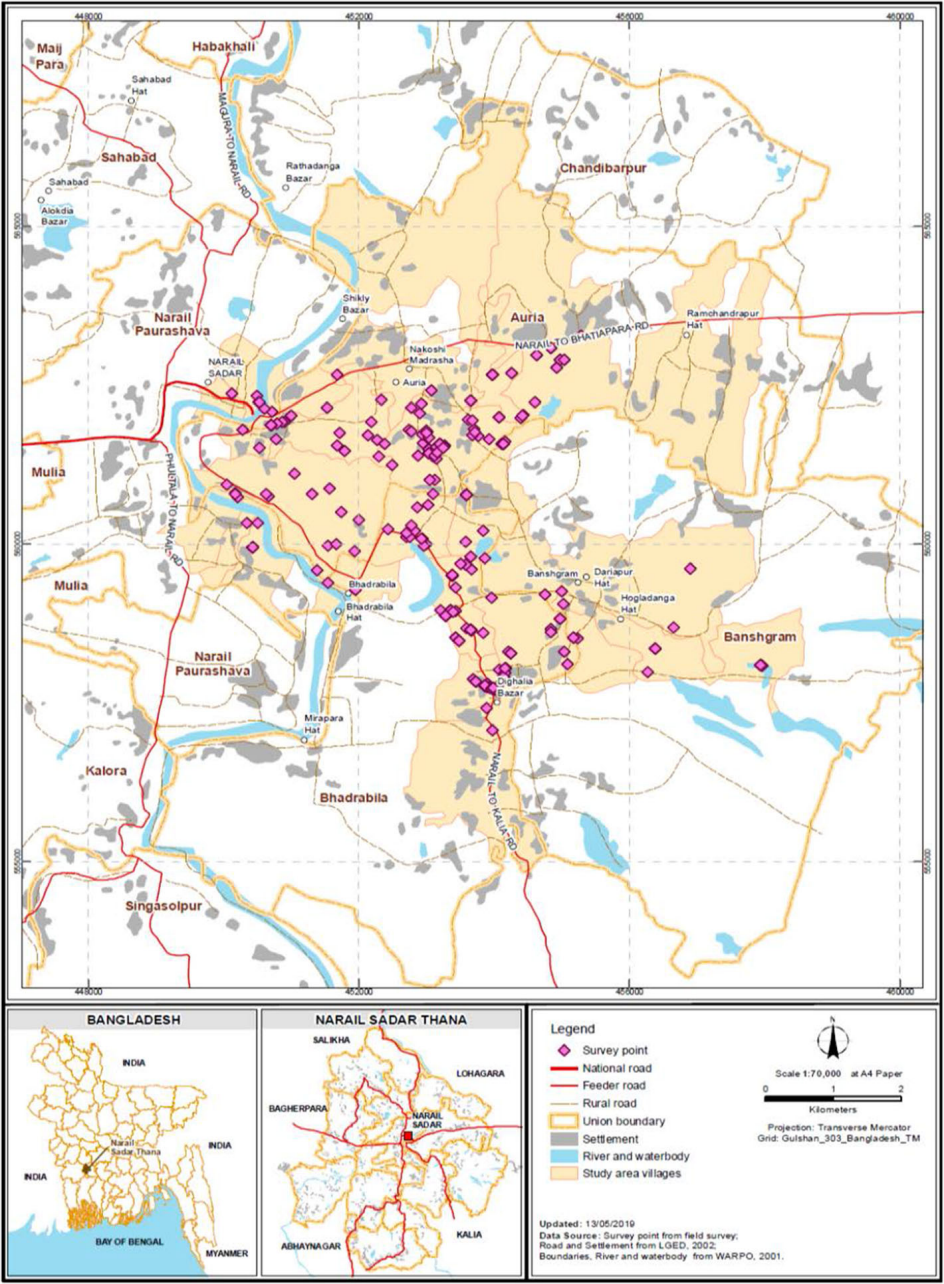
Location of the study area with survey location point

Bangladesh is a nation of 167 million individuals separated into 64 districts [21]. Adult participants aged 18 to 85 years were chosen from the Narail district, which is located about 200 km south-west of Dhaka, the capital city of Bangladesh, between July and August of 2018. The examination region incorporating a specific geographic zone and 300 survey points of information are shown in detail in Fig. 1.

**Box 1:** Individual items in the 19-item modified WHOQOL scale, including domain names and item numbers (in brackets)

**Overall quality of life (1)** **Satisfaction of health (2)**			
**Physical domain:** **Five items** Pain (3) Dependence of medical aids (4) Energy (10) Mobility (15) Activities of daily living (17)	**Psychological domain:** **Four items** Positive feeling (5) Concentration (7) Bodily image (11) Self-esteem (19)	**Social domain: Three items** Personal relationship (20) Sexual activity (21) Social support (22)	**Environmental domain:** **Five items** Security (8) Physical environment (9) Financial support (12) Accessibility of information (13) Leisure activity (14)
Item numbers shown in brackets (items 16 and 18 from physical; 6 and 26 from Psychological; 23, 24 and 25 from environmental domains), are deleted from the original WHOQOL-BREF scale.

### Sample size and statistical Power

A sample size of 300 was used. This is appropriate for a Rasch examination because large sample sizes can result in type 1 errors that falsely dismiss an item for not fitting the Rasch model [31]. A sample size of 300 is viewed as sufficiently substantial to ensure 99% confidence that the item difficulty would be within ±½ logit [32] .

### Sampling frame

The cross-sectional study recruited a multi-stage cluster random sample of 320 participant from the rural district Narail of Bangladesh in the period of July–August 2018. Information was collected from three unions (the smallest rural administrative unit) out of nine unions, excluding the four which were selected previously from the 13 unions of Narail Sadar Upazilla [33]. The selected unions were Auria, Banshgram and Bhardabila. One village (the smallest territorial and social unit for administrative and representative purposes) from each of the selected unions, was randomly selected at the second level. The selected villages were Baliadanga, Fulshor and Rogunathpur. Two paras (further divisions of the village) from each selected village were then randomly selected at the third level. Altogether, 40 adults (18–59 years old) and 40 older adults (60–85 years old) from each village were interviewed. Three hundred and twenty participants were interviewed for data collection. Interviewers used mobile data collection platform CommCare on their android phone to collect data from the respondents. To mitigate the effect of selection bias, 300 respondents were used with an equal proportion of adults and older adults, further partitioned into gender. This study excluded 20 participants randomly as 300 participants was deemed sufficient for the Rasch Measurement Theory.

### Data collection using CommCare and its advantage over using a printed questionnaire

Mobile data collection is a method employed to collect any qualitative and quantitative inputs via a mobile device (e.g. mobile phone, tablet, etc.). The introduction of mobile devices has streamlined data collection and made it more economical and less time consuming [34]. Other benefits include the minimising human error, speeding up reporting, being flexible in deploying programmatic changes, and providing accurate location information [35]. CommCare is a customisable and mobile platform that empowers non-developers to build mobile applications for data collection [36]. CommCare allows mobile applications to run offline and gather information transmitted to CommCareHQ when internet connectivity becomes accessible [37].

The current study followed a strict protocol to ensure a smooth launch when the application was finalised. The application was also thoroughly tested before training began [38]. We pilot tested the software with 30 people and found some minor problems associated with respondents not understanding the application correctly. These concerns were addressed through an upgraded version of the application which was then distributed for final data collection.

### Modified 19-item WHOQOL scale

Uddin et al. [30] validated WHOQOL-BREF scale and developed 19-item modified WHOQOL scale. Box 1 refers to individual items of the 19-item WHOQOL scale, including domain names and item numbers (in brackets). This consisted of two overall items, five items for the physical domain, four items for the psychological domain, five items for the environmental domain, and three items for social domain. Respondents were asked, during the previous 4 weeks, how satisfied they felt in each aspect of the domains. A Likert’ scale was used ranging from 1 to 5 where 1 designates ‘very dissatisfied’, 2 defines ‘dissatisfied’, 3 entitles ‘neither satisfied nor dissatisfied’, 4 levels ‘satisfied’ and 5 means ‘very satisfied’. Scores of each item were initially analysed using SPSS version 25, and while the Rasch analysis utilised in this investigation was conducted using the software package RUMM 2030 [39]. Since trained interviewers performed face-to-face interviews using an android phone to collect the data, there was no missing data. The only exception was item 21 (*sexual activity*). The respondents were reluctant to answer this item. This will be discussed in more detail in the discussion section. Negatively phrased items (*Pain (3) and Dependence of Medical Aids (4))* were recoded to convert into positively framed questions.

### Outcome measures

The 19-item modified WHOQOL scale is the main outcome measure for assessing general QoL using Rasch analysis. Demographic details were collected for age, gender, level of education and socio-economic conditions.

### The Rasch model

The Rasch model was named after Danish mathematician Georg Rasch [40]. It is a unique approach of mathematical modelling based on a latent trait and does probabilistic conjoint additivity (conjoint means the measurement of persons and items on the same scale, additivity is the equal-interval property of the scale). The Rasch model states that the probability of a person giving a certain answer, or endorsing an item, is a logistic function of the difference between the person’s ability and the item’s difficulty. Two versions of the Rasch model are available: dichotomous [41] and polytomous [42]. The polytomous Rasch model was utilised in this investigation. The Rasch model has two types, the Rasch Rating Scale Model [42] and the Partial Credit Model [43], which may be used with polytomous data. The partial credit model is the default within RUMM2030, placing no constraints on threshold parameters and allowing them to vary by item [44]. The likelihood ratio test, which is available in the RUMM2030 software, evaluates the unrestricted parameterization (partial credit model) against the reparameterization. A non-statistical result shows that the concept of a rating scale should be used, while statistically significant results indicate that the partial credit model is to be used [45]. The analysis was performed and identified a significant outcome that supports the use of the partial credit model.

The Rasch analysis generates several hypotheses that should be assessed to guarantee an instrument has Rasch properties. The most commonly evaluated Rasch properties are a) unidimensionality, b) local independence and c) invariability [46]. Unidimensionality is used to determine whether all information variability can be explained by a single latent trait. Local independence is the assumption that the response to one item should not lead to a response to another item [47]. That ensures that there is no significant correlation coefficients among items after extraction of the unidimensional latent variables, i.e. the residual correlations should be approximately zero [48]. The invariance criterion implies that generally an instrument should function in the same way for all individuals [49]. In the study overall fit of the Rasch model is assessed using item-trait interaction statistics [50]. This is reported in RUMM2030 as Chi-square statistics and should be non-significant (following a Bonferroni correction for the number of items in the scale), which is required for the data to be fit with the Rasch properties [51]. A significant value indicates that the hierarchical ordering of items varies across the trait, which compromise the required properties of invariance. Item-person interaction statistics are also considered (for items and persons), that are exhibited as z-statistics, where a perfect fit have a mean of 0 and standard deviation (SD) of 1. A fit residual SD value of 1.5 or less is commonly considered to indicate an acceptable fit. Individual item fit residuals (IFR) and person fit residuals (PFR) are also examined using residuals as well as Chi-square statistics. IFR and PFR satisfy the Rasch criteria when the residuals value falls within the range ± 2.5 and displays a non-significant chi-square value [52]. Residuals values below − 2.5 indicate overfit or redundancy and above 2.5 indicate an underfit item.

A “threshold” parameter is characterized by two response options where either response is equally likely. Disordered thresholds demonstrate that the respondents have difficulty differentiating between the response’s choices. Disordered thresholds result in item misfit and can be redressed by combining two neighbouring response options [53]. Unidimensionality suggests that the scale estimates just a single trait [54]. Following a principal component analysis (PCA) of the residuals, correlations between items and first PCA factors are used to define two subsets of items. The independent t-test is then used to assess the discrepancy in person estimations between the two subsets, with a non-significant result or the binomial distributions confidence interval’s lower bound overlap by 5% showing no evidence of multidimensionality [55]. The person-item residuals correlation matrix is used to determine whether there is any local dependency between the items, and correlations less than 0.3 are generally considered to be acceptable [45]. Differential item functioning (DIF) happens when two groups with a similar trait level react differently to an item [56, 57]. Age, sorted as either adult (18 to 59 years) or older adult (60 to 85 years), sex (male or female), education (no education or at least primary) and socio-economic conditions low (insufficient funds most/some of the time) and high (balance/sufficient funds all the time)) were used as DIF factors. Rasch examination gives a marker of reliability. In RUMM 2030, this is given by the person separation index (PSI) [58]. The PSI is comparable to Cronbach’s alpha (CA); a value near 1 shows high internal consistency and a value under 0.7 demonstrates model low scale reliability [59]. Scale targeting is assessed graphically by examining the person-item distribution, which shows the individual scores and the positioning of the item on the underlying trait. In a well-targeted scale, items would span the full range of individual scores. For a well-targeted measure (not too easy, not too hard), the mean location for persons would be a value around zero. A positive mean value for persons indicates that the sample is located at a higher level QoL than the average of the scale, while a negative value suggests the opposite [60].

## Results

Table 1 describes the socio-demographic characteristics of the participants by gender (male vs female). The mean (standard deviation (SD), range) age of the participants was 52.0 years (15.6, 18–85). Forty-five percent of the participants did not have any formal education and only 1.3% attained a bachelor’s degree or above. More than 70% of the respondents had some financial instability.

**Table 1:** Sociodemographic characteristic of the study participants by gender in the Narail district of Bangladesh

Characteristic	Total, ***N*** = 300	Female, ***N*** = 150	Male, N = 150	***P*** value *
	Number (%)	Number (%)	Number (%)	
**Age group**				
Adult (18–59 years)	150 (50)	75 (50)	75 (50)	
Elderly (60–85 years)	150 (50)	75 (50)	75 (50)	
**Level of education, number of years schooling**				
No education	135 (45)	80 (53)	55 (37)	0.004
Primary (1–5)	80 (27)	36 (24)	44 (29)	
Secondary (6–9)	64 (21)	31 (21)	33 (22)	
SSC or HSC Pass (10–12)	17 (6)	3 (2)	14 (9)	
Degree or equivalent (13–16)	4 (1)	0	4 (3)	
**Socio-economic condition**				
Insufficient funds most of the time	97 (32)	62 (41)	35 (23)	0.808
Insufficient funds some of the time	124 (41)	50 (33)	74 (49)	
Balance	76 (25)	37 (25)	39 (26)	
Sufficient funds most of the time	3 (1)	1 (1)	2 (1)	

Level of education: no education (: did not have any formal education), primary (1 to 5 years of schooling),; Secondary: (6 to 9 years of schooling); SSC (Secondary School Certificate) (complete 10 years of schooling through a national level of examination); HSC (Higher Secondary Certificate) (complete 12 years of schooling); Degree or Equivalent: Hons or Master’s complete 16 years of schooling) *pairwise comparison tested between the education level and socioeconomic status by gender.

**Fig. 2:**
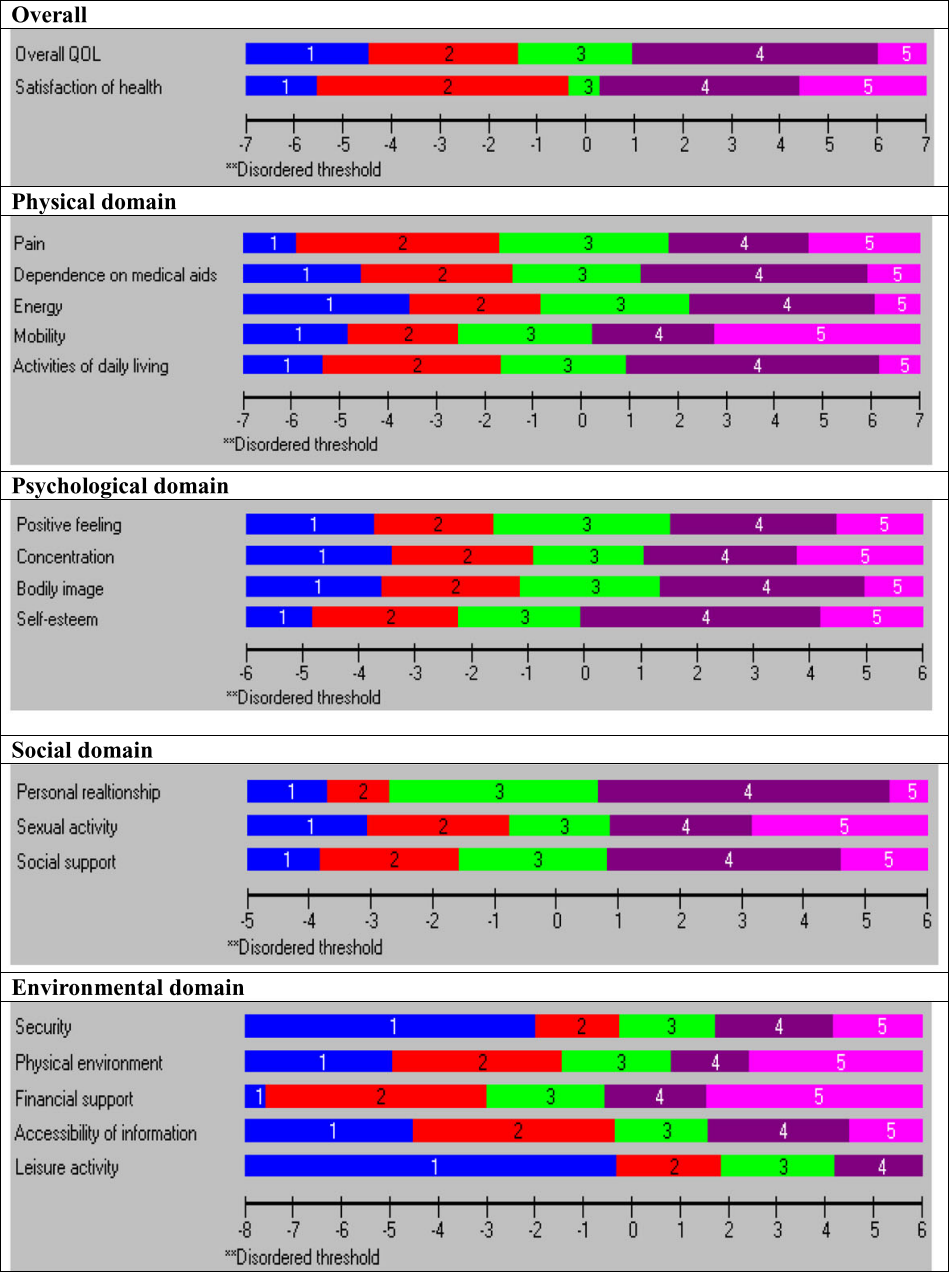
Threshold maps of the 19-item modified WHOQOL scale

The first two items of the 19-item WHOQOL (overall QoL and general health) scale appeared to represent perfect fit and ordered thresholds (Table 2 & Fig. 2). Hence, these two items were not considered for any part of the domains and no further examination was performed. The following sub-sections discuss the results of the validation sample for four underlying domains.

**Table 2:** Performance of the four domains of 19-item modified WHOQOL scale using Rasch model adjustment (sample size, *n* = 300)

	Data on Validation Process					Summary of overall model fit statistics for each domain (validation on modified instrument)	
	Location	SE	Residual	χ^**2**^	***P*** value		
**Overall**							
Overall QoL (1)	0.29	0.10	0.45	2.48	0.63		
General Health (2)	−.029	0.09	0.59	2.99	0.25		
**Physical domain**						**Unidimensionality test**	**3.7% CI (1.2–6.1)**
Pain (3)	−0.30	0.11	−1.82	8.42	0.07	Person separation index	0.873
Dependence of medical aids (4)	0.24	0.11	1.73	5.83	0.21	Cronbach’s Alpha	0.881
Energy (10)	1.02	0.10	−0.65	3.37	0.49	Chi-square (Degrees of freedom)	32.13 (20)
Mobility (15)	−1.04	0.10	−0.38	5.88	0.20	Probability value	0.0418*
Activities of daily living (17)	0.08	0.11	−0.76	8.63	0.07	Items fit residual, mean (SD)	−0.17 (1.38)
						Persons fit residual, mean (SD)	−0.42 (1.05)
**Psychological domain**						**Unidimensionality test**	**4.3% CI (1.9–6.8)**
Positive feeling (5)	0.18	0.10	0.48	2.52	0.64	Person separation index	0.739
Concentration (7)	0.14	0.09	−0.32	2.04	0.73	Cronbach’s Alpha	0.746
Bodily image (11)	0.40	0.10	0.57	3.23	0.52	Chi-square (Degrees of freedom)	14.93 (16)
Self-esteem (19)	−0.72	0.10	−0.39	7.15	0.13	Probability value	0.529
						Items fit residual, mean (SD)	0.08 (0.51)
						Persons fit residual, mean (SD)	−0.42 (1.01)
**Social domain**						**Unidimensionality test**	**3.3% CI (0.9–5.8)**
Personal relationship (20)	−0.08	0.13	−0.42	9.51	0.05	Person separation index	0.650
Sexual activity (21)	0.63	0.10	0.22	2.12	0.73	Cronbach’s Alpha	0.670
Social support (22)	0.01	0.11	0.49	0.98	0.91	Chi-square (Degrees of freedom)	12.62 (12)
						Probability value	0.397
						Items fit residual, mean (SD)	0.10 (0.46)
						Persons fit residual, mean (SD)	−0.44 (0.92)
**Environmental domain**						**Unidimensionality test**	**5.0% CI (2.5–7.5)**
Security (8)	0.96	0.08	−0.50	1.12	0.89	Person separation index	0.753
Physical environment (9)	−0.70	0.09	−0.43	2.98	0.56	Cronbach’s Alpha	0.781
Financial support (12)	−2.31	0.09	0.93	5.70	0.22	Chi-square (Degrees of freedom)	22.01 (20)
Accessibility of information (13)	0.37	0.10	−0.78	5.17	0.27	Probability value	0.339
Leisure activity (14)	1.68	0.10	0.78	7.03	0.13	Items fit residual, mean (SD)	−0.01 (0.79)
						Persons fit residual, mean (SD)	−0.32 (0.96)

Person separation index and Cronbach’s Alpha assess the reliability of the scale: **χ**^**2**^ Chi-square value CI: Confidence interval, SE: Standard Error, SD: Standard deviation

Probability of Chi-square value should be higher than .05, Because the number of items of the physical domain is five, so with Bonferroni corrected p-values for the domain is (.05/5 = 0.01). Any p- value > 0.01 will be considered non-significant at 0.05 level for physical domain.

### Physical domain subscale

The PSI for physical domain of five items was 0.873 and CA was 0.881, indicating that the reliability of the physical domain was good (Table 2). All items showed ordered thresholds (Fig. 2); for the response options based on a five-point Likert-type scale (0, 1, 2, 3, 4). Overall, the domain showed good fit, as evident with item-trait interaction statistics (${\upchi}_{(20)}^2$ =32.13, *p* = 0.041) (Table 2). Comparing the *p*-value 0.01 after Bonferroni correction, (0.05/5 = 0.01) the physical domain had non-significant Chi-square *p-*values. In addition, standardised IFR statistics (mean = − 0.17, SD = 1.38), PFR (mean = − 0.42, SD = 1.05) were within acceptable range and all items in the physical domain had non-significant Chi-square *p-*values (Table 2). There was no evidence of DIF for the socio-demographic variables (age and gender, educational attainment and socio-economic conditions) (Table 3). PCA of the residuals was conducted to identified positive and negative loading of items on the first extracted component. Comparison of the person estimates generated from these two subsets indicated that only 3.7% (95% Confidence Interval: 1.2 to 6.1%) of cases were statistically significantly different (Fig. 4), that shows unidimensionality of the domain. The domain also had no local dependency as no correlation coefficient between the items is greater than 0.3.

**Table 3:** DIF on age, gender, educational attainment and socio-economic conditions of the four domains of 19-item modified WHOQOL scale

	DIF on Age			DIF on Gender			DIF on Education			DIF on Socio-economic Status		
	MS	F	P*	MS	F	P*	MS	F	P*	MS	F	P*
**Physical domain**												
Pain (3)	0.72	1.28	0.26	1.24	2.24	0.14	1.03	1.84	0.18	0.01	0.02	0.89
Dependence of medical aids (4)	1.45	1.46	0.23	2.59	2.56	0.11	2.22	2.50	0.05	0.51	0.51	0.48
Energy (10)	0.79	1.03	0.31	2.30	3.85	0.05	3.34	4.37	0.06	0.00	0.00	0.98
Mobility (15)	3.44	4.43	0.04	0.00	0.00	0.95	2.86	3.68	0.06	2.50	3.52	0.05
Activities of daily living (17)	0.17	0.24	0.62	0.68	0.94	0.33	1.46	2.03	0.16	1.61	2.24	0.14
**Psychological domain**												
Positive feeling (5)	0.17	0.22	0.64	0.08	0.11	0.75	0.15	0.19	0.67	0.12	0.15	0.70
Concentration (7)	1.36	1.89	0.17	0.02	0.03	0.85	0.59	0.82	0.37	1.17	1.62	0.21
Bodily image (11)	1.74	2.20	0.14	0.90	1.13	0.29	0.89	1.12	0.29	0.45	0.56	0.45
Self-esteem (19)	0.22	0.33	0.57	0.67	0.96	0.33	0.01	0.02	0.89	0.03	0.04	0.84
**Social domain**												
Personal relationship (20)	1.08	1.85	0.18	0.04	0.08	0.78	0.01	0.01	0.91	0.09	0.16	0.69
Sexual activity (21)	1.81	2.68	0.10	0.22	0.31	0.58	0.17	0.26	0.61	1.10	1.60	0.21
Social support (22)	0.28	0.38	0.54	0.64	0.89	0.35	0.02	0.03	0.86	1.21	1.69	0.20
**Environmental domain**												
Security (8)	0.99	1.29	0.26	1.32	1.72	0.19	1.88	2.47	0.12	0.02	0.02	0.88
Physical environment (9)	2.76	3.65	0.06	0.10	0.14	0.71	2.35	3.10	0.08	4.05	5.54	0.06
Financial support (12)	1.97	2.29	0.13	0.06	0.07	0.79	0.56	0.64	0.42	5.17	6.15	0.05
Accessibility of information (13)	2.12	2.93	0.09	0.03	0.04	0.85	0.02	0.03	0.87	0.55	0.77	0.38
Leisure activity (14)	0.03	0.03	0.85	5.13	6.08	0.01	0.01	0.01	0.93	0.21	0.25	0.62

*P ≤ 0.01 indicates significant difference after Bonferroni corrections for five items for the physical and the environmental domain. (any *p* values > 0.01 will be considered non-significant at 0.05 level)

***MS*** Mean square and ***F*** F test value*.*

### Psychological domain subscale

The PSI for the psychological domain of four items was 0.739 and CA was 0.746, indicating that the reliability of the psychological domain was acceptable (Table 2). All items showed ordered thresholds (Fig. 2). Overall, the domain showed good fit, as evidenced by item-trait interaction statistics (${\upchi}_{(16)}^2$ =14.93, *p* = 0.529), IFR (mean = − 0.08, SD = 0.51) and PFR (mean = − 0.42, SD = 1.01) (Table 2). All individual item fit statistics were non-significant (Table 2). There was no evidence of DIF for the socio-demographic variables (age groups, gender, educational attainment and socio-economic conditions) (Table 3). There was no indication of multidimensionality with independent t-tests, contrasting person-location estimates from two subsets identified using PCA of the residuals, suggesting only 4.3% (95% Confidence Interval: 1.9 to 6.8%) statistically significant different (Fig. 4). There were no correlation coefficients above 0.30 on the person-item residual correlation matrix, indicating no local dependency of the items of psychological domain.

### Social domain subscale

Initially, the social domain had insufficient reliability (PSI = 0.594) and item-trait interaction statistics was (${\upchi}_{(12)}^2$ =18.91, *p =* 0.090) (data were not shown in the table). Because of missing data, RUMM 2030 could not produce CA. Initially, 88 participants with missing data were removed from item 21 (*sexual activity*) and the domain was reanalysed using the remaining 212 participants. Without missing data social domain item-trait interaction statistics ((${\upchi}_{(12)}^2$ =12.62, *p =* 0.397), PSI = 0.650 and CA = 0.669) (Table 2) represent a below acceptable reliability. No serious misfit was observed for both persons and items as evident by IFR (mean = 0.10, SD = 0.46) and PFR (mean = − 0.44, SD = 0.92). The social domain had no local dependency, no disordered thresholds (Fig. 2), no DIF by age group, gender, educational attainments and socio-economic status and no evidence of multidimensionality (Fig. 4). As the domain had only three items, it was assessed that reliability for Rasch assumptions could be deemed ‘reasonable’.

### Environment domain subscale

**Fig. 3:**
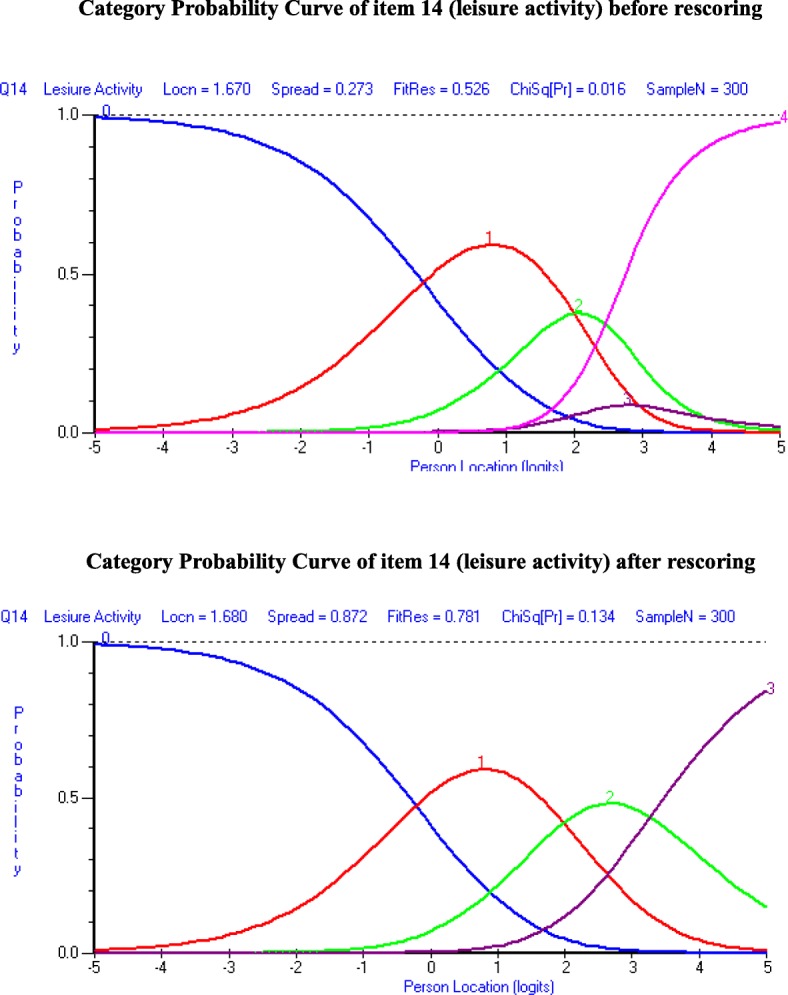
Category Probability Curve of the item 14 (Leisure activity) before and after rescoring

**Fig. 4:**
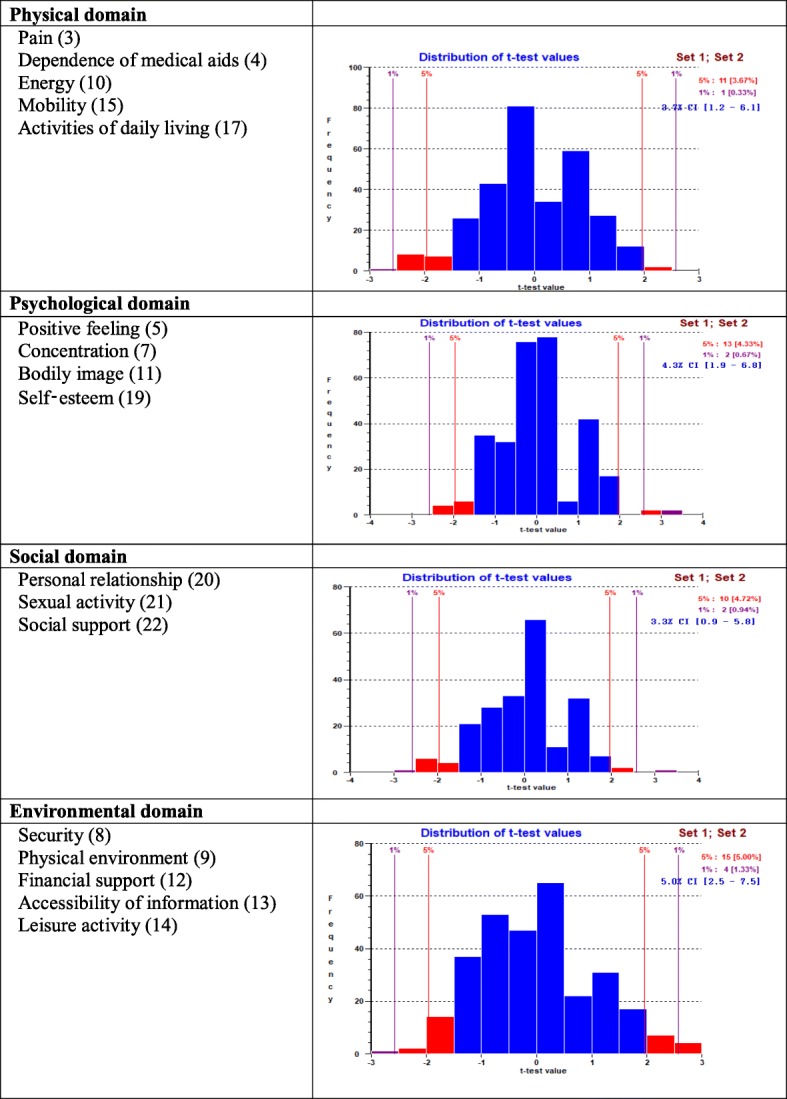
Dimensionality testing of the 19-item modified WHOQOL scale

At the beginning, the environmental domain showed acceptable fit (${\upchi}_{(20)}^2$ =28.29, *p* = 0.102, PSI = 0.754). PFR and IFR residuals statistics were within an acceptable range. However, a disordered threshold was observed for item 14 (*leisure activity*) (Fig. 3, top part). Respondents were experiencing issues discriminating category response options 3 and 4, indicating problems with the categorisation of the response options across the trait. To make the environmental domain more fitting, the two middle adjacent response options 3 and 4 put together, which reduced one response option from the original five response options and resolved the disordered threshold for item 14 (Fig. 3, bottom part). Overall Chi-square interaction statistics (${\upchi}_{(20)}^2$ =22.01, *p* = 0.339, IFR (mean = − 0.01, SD = 0.79), and PFR (mean = − 0.32, SD = 0.96)) was strengthened by the rescoring of the item. The socio-demographic variables (different age/sex distributions, educational attainment and socio-economic conditions) have not been detected by DIF (Table 3). The PSI value was 0.753 and CA = 0.781, indicating that environmental domain reliability was good; all individual item fit statistics were non-significant (Table 2), and all items had ordered thresholds (Fig. 2). The unidimensionality of the five-item environmental domain was supported by the independent t-tests comparing the person estimates with the PCA of the residuals; the study findings indicated that only 5.0% (95% CI 2.5 to 7.5%) cases showed statistically significant differences (Fig. 4). The person-item residual correlation matrix had no correlation coefficients above 0.30, suggesting no local dependency on the items*.*

### Targeting of each of the four domains of the 19-item modified WHOQOL scale

**Fig. 5:**
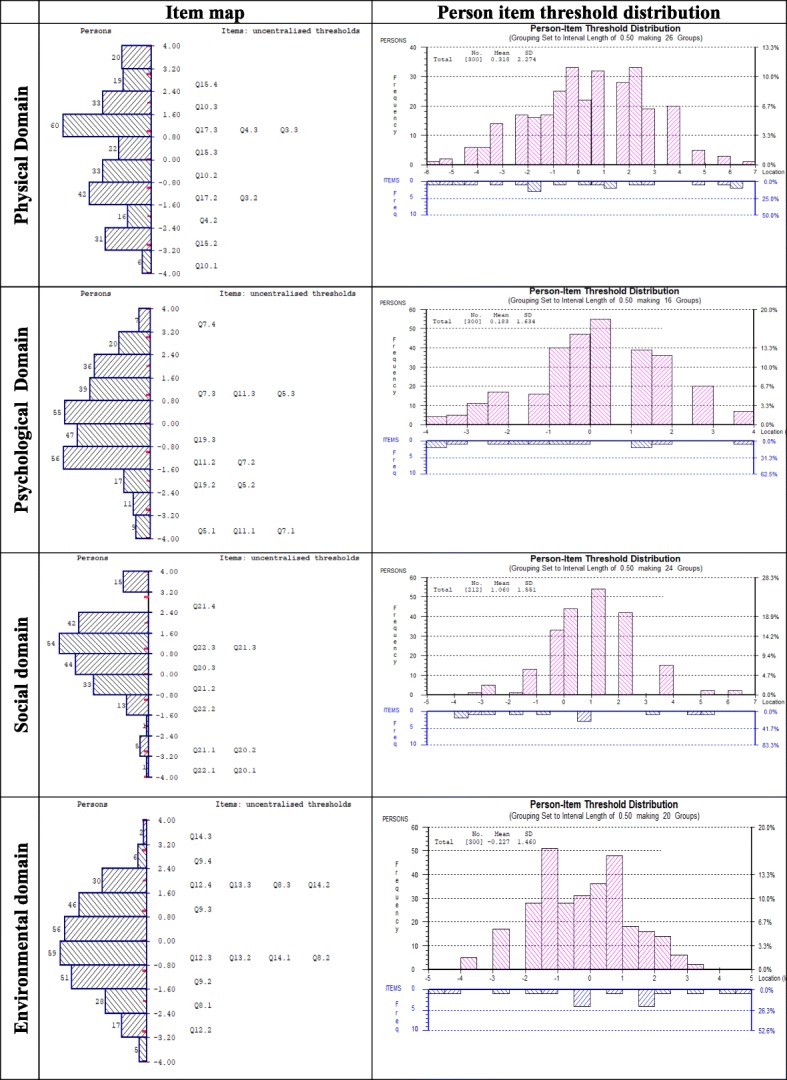
Item map for the person-item threshold distribution of the 19-item modified WHOQOL scale

Figure 5 presents the item map for the person-item threshold distribution of the four domains, showing targeting of the 19-item WHOQOL scale. The person distribution is shown in the top half and the item thresholds in the bottom half. The overall mean person logit for the physical domain was 0.318, the psychological domain was 0.183, the social domain was 1.060 and the environmental domain was − 0.227, suggesting well-targeted persons and items for each of the domains except the social domain. A positive mean value for physical, psychological and social domains would indicate that the sample was located at a higher level (e.g., QoL) than the average of the scale, while a negative value for the environmental domains suggests a slightly lower level of QoL. Overall, the scale was not too difficult to endorse.

## Discussion

Quality of life screening instruments are now widely used in both clinical practice and research. The purpose behind this article was to evaluate the Rasch measurement properties of the 19-item modified WHOQOL scale (which was developed from 26-item WHOQOL-BREF), to assess the psychometric properties of the scale as a measure of QoL. This paper uses a Rasch examination to explore several issues relating to the validation of the 19-item WHOQOL scale. To date, this could be deemed as the first study using the Rasch model to examine the psychometric properties of the 19-item modified WHOQOL scale in a typical healthy people in rural Bangladesh. The article also checks the integrity of the category scorings used in the scale; individual item fit statistics and an assessment of DIF according to an age-sex distribution, education attainment, and socio-economic status.

The 26-item WHOQOL-BREF scale facilitated psychometric evaluations in rural Bangladesh and developed a 19-item WHOQOL scale that was a substantial instrument to measure the QoL of rural Bangladesh [30]. In any case, further approval is required to affirm its utilisation in other rural study settings with healthy population. Overall, four domains (physical, psychological, social and environmental) of the 19-item scale demonstrates good reliability (except social domain), no misfit items, and no multidimensionality. All items within the four domains have ordered thresholds except one item in the environmental domain (*leisure activity),* where respondents could not distinguish between the two response formats *moderate and mostly*. After rescoring the items by merging the two categories *moderate and mostly* to one category the problem of disorder thresholds was resolved.

The reliability for the physical and the psychological domains were superior to earlier validated physical and psychological domains [30]. For the social domain the reliable estimates between the present analysis and the previous study are essentially the same [30]. For the environment domain, this analysis found that reliability was only marginally below the previous estimates [30]. Hence, the present investigation further supports our contention that the 19-item WHOQOL scale is a useful instrument for estimating QoL in rural Bangladesh.

There has been controversy over the DIF associated with Gender in QoL assessment [61]. There is a stable nonlinear association between age and person QoL in various cross-sectional epidemiologic investigations [62, 63]. The validated domain scale exhibited no DIF on sex (male and female) and age (adult and older adults) and is in line with various population studies [30, 61–64]. Higher levels of QoL are present among individuals with higher educational attainment compared with lower educational attainment [65, 66]. Although there may still be an association between SES and QOL such as low socioeconomic status associates with a lower level of QoL [67], the lack of DIF means that items function the same way with regards to their psychometric properties, irrespective of SES group.

When evaluating any scale using a CCT approach, there is an assumption that each item has equal weight to the final score. So, the evaluation focuses only on the total score but not on the individual item score. Several previous studies in Bangladesh were unable to delete any individual items because the studies had used CCT methods to validate the WHOQOL-BREF scale [24, 25, 68]. A comparison of this study with previous studies [24, 25, 68], is therefore somewhat limited. However, Uddin et al. used Rasch analysis and developed a 19-item modified WHOQOL scale that would be suitable for rural Bangladesh. The current study further confirms the 19-item scale that (Uddin et al. [30] proposed) provides adequate fit in yet another rural setting in Bangladesh.

The research revealed further noteworthy issues regarding the social domain. The social domain contains just three items and one of the items related to the respondent’s sexual life, which may not be significant to older, widowed and unmarried individuals. Despite this, some respondents may have a sex life outside of marriage; however, due to social pressure they do not disclose this to interviewers. Previously, Gott et al. [69] and Uddin et al. [30] raised this issue in their published papers, however, no suitable option has been proposed in the literature to date as issues associated with determining answers in religious countries such as Indonesia, Bangladesh and Middle-eastern states appear to be complex and very challenging. Uddin et al. [30] has proposed mature aged female interviewers need to be assigned to female participants while older-aged males should be assigned to male participants for the sensitive question. However, these approaches did not improve the respondent reactions to the item related to their sexual life, where 88 respondents out of 300 were still not ready to give an answer on that item (older, widowed and unmarried participants regardless of gender). In that respect, the elective methodology did not help in religious based nations. Sensitivity to answer the item may reduce the social domain reliability. However, similar result was found from the WHOQOL group documenting Cronbach’s alpha of 0.70 from their total group analysis, and 15 out of 24 centres have CA < 0.70 [20]. The targeting of the sample was less than the desired value in the social domain. In specific, an item related to sexual life (item 21) where comparatively lacking responses have been reported. However, after excluding missing data, it has a reasonable degree of precision. Nevertheless, testing of better-targeted populations in the multicentre study, with higher levels of education, would further support the better targeting of the social domain. Future studies should attempt to improve the above limitations in other rural area rewording item 21.

The Rasch examination contributes valuable information on four domains of the 19-item WHOQOL scale applied amongst the general rural population of Bangladesh. Interviewers used mobile data collection platform CommCare to collect data from the respondents to minimising human error and speeding up reporting [38]. Moreover, the 19-item WHOQOL scale can be made openly accessible in any health care setting as well as on the web. Given its brevity and straightforwardness in both online and paper format, the 19-item WHOQOL scale might be a support to individuals searching for a self-detailed assessment of individual QoL.

The potential limitations of this examination relate to its reliance upon single-event information from individuals in another rural area of Bangladesh. While we have endeavoured to validate the modified 19-items WHOQOL scale in the rural region of Narail. Replication studies with large populated samples of Bengali speakers may improve generalisation. The problem of fit statistics in the Rasch analysis is that the larger the sample size, the higher the probability of detecting deviations from the Rasch model [70, 71]. However, there are no exact recommendations for sample size when performing Rasch Measurement Theory and the power to detect misfit and bias increases with sample size [72]. While we have used sample size of 300 which is more preferred one [70].

## Conclusion

As far as we know, this research is the first one to apply Rasch methods in the healthy rural population of Bangladesh to validate 19-item modified WHOQOL scale. The study suggests there is sufficient evidence to recommend the utilisation of the modified 19-item WHOQOL scale to measure QoL in rural Bangladesh. The four domains of the modified 19-item WHOQOL scale seemed to contain no DIF on age, sex, educational attainment, and different socio-economic conditions. Each domain of the 19-item WHOQOL scale was unidimensional and none had any local dependency. Such findings indicate that the 19-item WHOQOL scale is appropriate to determine QoL in rural Bangladesh. Future research may continue to assess the 19-item WHOQOL scale and further assess the items of the social domain, particularly in clinical settings environment. The cross-cultural validity of the 19-item WHOQOL scale may be further explored in the multi-centre assessment of QoL in various rural areas of Bangladesh to explore the domains effect on QoL in (HR-QoL) assessments.

## Data Availability

The datasets used and/or analysed during the current study are available from the corresponding author on reasonable request.

## Abbreviations

WHOQOL-BREF: The World Health Organisation Quality of Life Instruments, Short Form
QoL: Quality of life
PSI: Person separation index
CTT: Classical test theory
DIF: Differential item functioning
CA: Cronbach’s Alpha
IFR: Item fit residuals
PRF: Person fit residuals
PCA: Principal component analysis
HR-QoL: Health related quality of life
